# Retracted: Ferroptosis-Related lncRNA for the Establishment of Novel Prognostic Signature and Therapeutic Response Prediction to Endometrial Carcinoma

**DOI:** 10.1155/2022/9829634

**Published:** 2022-12-27

**Authors:** BioMed Research International

*BioMed Research International* has retracted the article titled “Ferroptosis-Related lncRNA for the Establishment of Novel Prognostic Signature and Therapeutic Response Prediction to Endometrial Carcinoma” [1] due to concerns that the peer review process has been compromised.

Following an investigation conducted by the Hindawi Research Integrity team [2], significant concerns were identified with the peer reviewers assigned to this article; the investigation has concluded that the peer review process was compromised. We therefore can no longer trust the peer review process and the article is being retracted with the agreement of the editorial board.

The authors agree to the retraction.
